# SterylAcetyl Hydrolase 1 (BbSay1) Links Lipid Homeostasis to Conidiogenesis and Virulence in the Entomopathogenic Fungus *Beauveria bassiana*

**DOI:** 10.3390/jof8030292

**Published:** 2022-03-11

**Authors:** Yue-Jin Peng, Hao Zhang, Ming-Guang Feng, Sheng-Hua Ying

**Affiliations:** Institute of Microbiology, College of Life Sciences, Zhejiang University, Hangzhou 310058, China; 11907003@zju.edu.cn (Y.-J.P.); 22007020@zju.edu.cn (H.Z.); mgfeng@zju.edu.cn (M.-G.F.)

**Keywords:** steryl acetyl hydrolase 1, lipid homeostasis, entomopathogenic fungus, development, virulence

## Abstract

*Beauveria bassiana*, as a well-studied entomopathogenic fungus, has a great potential for the biological control of insect pests. Lipid metabolism has been linked to the life cycle of *B. bassiana*; however, the underlying mechanisms remain unknown. In this study, a homolog of yeast steryl acetyl hydrolase 1 (Say1) was functionally characterized. The loss of *B. bassianaSAY1* (*BbSAY1*) impaired the lipid homeostasis in conidia, with a significant reduction in oleic acid content. The Δ*Bbsay1* mutant strain displayed anelevated accumulation of lipid bodies and aweakened membrane permeability. As for phenotypic aspects, gene loss resulted in significant defects in germination, conidiation, and virulence. Our findings highlight that Say1, involved in lipid homeostasis, contributes to the cytomembrane integrity, development, and virulence in *B. bassiana*.

## 1. Introduction

*Beauveria bassiana* is one of the most prevalent insect pathogens in the eco-system and has great potential for the biological control of pests [1,2]. Conidia, the major form of infectious cells, germinate and ingress the host body through the trans-cuticular route. In the host hemocoel, fungal cells undergo replication and overcome the host defense [3,4]. After the host dies, hyphae grow outside the host body and produce a lot of conidia on the cadaver. The newly-born conidia will initiate the subsequent infection cycle when encountering the sensitive hosts [5].

In *B. bassiana*, conidia form on the ‘zig-zag’-shaped conidiophores and accumulate a plethora of fatty acids (FAs) and lipids [6,7]. The homeostasis of lipid metabolism is finely tuned by a series of processes of synthesis and degradation. FAs act as the synthetic precursors of storage lipids and membranes [8] and can be recycled from the degradation of lipids and membranes [9]. Oleic acid (OA) is an important unsaturated FA in fungal cells and is synthesized through the OLE pathway, in which the Δ9-fatty acid desaturase gene (Ole1) catalyzes the desaturation of palmitic acid (PA), critical for the synthesis of unsaturated fatty acids [10]. HapX represents a family of basic leucine zipper (bZIP) transcription factors, which are indispensable for iron acquisition in fungi [7,11]. In *B. bassiana*, the OLE pathway also contributes to conidial storage of OA and is transcriptionally regulated by HapX (BbHapX). In addition, BbHapX has a greater influence on lipid homeostasis than BbOle1 [7]. However, the mechanisms underlying the lipid homeostasis remains enigmatic. In budding yeast, steryl acetyl hydrolase 1 (Say1) is required for lipid homeostasis [12]. We supposed that the ortholog of yeast Say1 might be required for lipid homeostasis in *B. bassiana*.

In this study, we functionally characterized Say1 in *B. bassiana* by constructing the gene disruption and complementation mutant strains. Our data revealed that *BbSAY1* contributes to conidial lipid reserves, formation, and virulence.

## 2. Materials and Methods

### 2.1. Microbial Strains and Cultivation

The wild-type strain of *B. bassiana* ARSEF2860 was cultured on Sabouraud dextrose agar (SDAY: 4% glucose, 1% peptone, 1.5% agar, and 1% yeast extract) for routine maintenance. *Escherichia coli* DH5α (Invitrogen, Carlsbad, CA, USA) for plasmid proliferation was cultured in Luria-Bertani (LB) medium with appropriate antibiotics. A yeast extract broth (*w*/*v*: 0.5% sucrose, 1% protein, 0.1% yeast extract, 0.05% MgSO_4_, and 1.5% agar) was used to culture *Agrobacterium tumefaciens* AGL-1, which was used in fungal transformation. Czapek-Dox agar (CZA) (3% sucrose, 0.3% NaNO_3_, 0.1% K_2_HPO_4_, 0.05% KCl, 0.05% MgSO_4_, and 0.001% FeSO_4_ plus 1.5% agar) was used as a chemical-defined medium.

### 2.2. Bioinformatic and Transcriptional Analyses of BbSay1 in B. bassiana

The protein sequence of *S. cerevisiae* Say1 (GenBank No.: DAA08353) was used as a query to search the potential ortholog in the *B. bassiana* genome [13]. After mapping the cDNA sequence of BbSay1, a complete open reading frame with upstream and downstream flanking sequences was obtained. Domain annotation was conducted through an online portal SMART (http://smart.embl-heidelberg.de/) (last accessed on: 9 September 2020). The ortholog of yeast Say1 in *B. bassiana* was determined with the method of *reciprocal best alignment* between two species [14]. After searching all the Say1-domain proteins in *B. bassiana*, their homologs in other fungi were retrieved via BLAST analyses and proofed via domain analysis. Tested fungal species included *Aspergillus nidulans* FGSC A4, *A. niger* CBS 513.88, *A. fumigatus* Af293, *Candida albicans* SC5314, *Cordyceps militaris* CM01, *Magnaporthe grisea* 70-15, *Metarhizium* *acridum*CQMa 102, *M. robertsii* ARSEF 23, *S. cerevisiae* S288C, and *Yarrowia* *lipolytica* CLIB122.

The transcriptional analyses of the Say1-domain protein genes were performed as the methods documented [15]. Conidia of the wild-type were cultured on a SDAY plate at 25 °C, and mycelia were sampled at 2, 3, 4, and 5 d. Total RNA was extracted from the mycelial samples with the RNAiso^TM^ Plus Reagent (TaKaRa, Dalian, China), and cDNA was reverse transcribed using the PrimeScript^®^ RT reagent Kit (TaKaRa). The resultant cDNA was used as templates to perform the qRT-PCR reaction on a Mastercycler^®^ EP Realplex (Eppendorf, Hamburg, Germany) cycler. All primers are listed in Table S1. The relative transcript level of each gene was calculated as the relative expression of different time points over 2 d using the fungal 18S rRNA as an internal reference and the 2^−^^ΔΔ^^Ct^ method [16].

Localization of BbSay1 was performed as described previously [17]. Primers sequences were listed in Table S2. The coding sequence of BbSay1 was amplified with primers PL1 and PL2, and then, it was fused to the green fluorescent protein gene (*GFP*) by ligating into the *Nco*I/*Eco*RI sites of pBMGB. The resulting vector was transformed into the wild-type strain. The transformant was screened on CZA plates with 200 µg/mL glufosinate and cultured on an SDAY plate at 25 °C. Fungal cells were sampled, and the fluorescent signals were observed under a laser scanning confocal microscope (LSM 710, Carl Zeiss Microscopy GmbH, Jena, Germany).

### 2.3. Gene Disruption and Complementation

The methods for gene disruption and complementation were the same as those described previously [18]. All primers arelisted in Supporting Information Table S2. Upstream (0.81 kb) and downstream (1.14 kb) flanking sequences of the *BbSAY1* open reading frame (ORF) were amplified by PCR with the paired primers P1/P2 and P3/P4, respectively, using genomic DNA as the template. The amplified PCR fragments were digested with *Eco*RⅠ/*Bam*HⅠ and *Xba*Ⅰ/*Hpa*Ⅰ, respectively, and then successively cloned into the *Eco*RI/*Bam*HI and *Xba*Ⅰ/*Hpa*Ⅰ sites of p0380-bar [19]. The resulting plasmid was named p0380-bar-Bbsay1 and used for gene disruption. The candidate disruptants were screened by PCR with the primers P5 and P6. To complement the gene loss, the intact BbSAY1 was ectopically integrated in the gene disruption mutant strain. The *BbSAY1* ORF plus 1.85 kb of upstream and 0.34 kb of downstream sequences was amplified with primers P7/P8, and the resultant fragment was recombined into the vector p0380-sur-gateway with a sulfonylurea resistance cassette [19], generating the plasmid p0380-sur-Bbsay1. The plasmids were transformed into the *B. bassiana* strain using an *Agrobacterium*-mediated transformation method. Putative disruption mutants were screened on CZA supplemented with phosphinothricin (200 μg/mL), and the complemented strains were screened on CZA plates with 10 mg/mL chlorimuron ethyl.

Southern blotting analyses were used to further confirm the transformants. Fungal genomic DNA (30 μg) digested by *Sac*Ⅰ/*Nco*Ⅰ were resolved on a 0.7% agarose gel and then electronically transferred to a Biodyne B nylon membrane (Gelman Laboratory, Shelton, WA, USA). Target fragments were hybridized with the probes prepared from the templates amplified with the primer pair P9/P10 and visualized with the DIG-High Prime DNA Labeling and Detection Starter Kit II (Roche, Penzberg, Germany).

### 2.4. Biochemical and Phenotypic Assays

The indicated strain was cultured on SDAY plates at 25 °C for 7 days, and the resultant conidia were suspended in a 0.02% Tween-80 solution. All phenotypes were compared among the wild-type, Δ*Bbsay1,* and complementation mutant strains with three parallel experiments [7,20].

#### 2.4.1. Quantification of Conidial FAs

Conidia (100 mg) were suspended in 10 mLCMW mixture (chloroform:methanol:water = 2:1:1, *v/v/v*) and placed at −20 °C for 2 h. Then, 3 mLCM mixture (chloroform:methanol = 2:1, *v/v*) was added and the conidial suspension stood for 1 h. The bottom layer was carefully collected and dried. FAs were methylated and analyzed on a Focus series gas chromatograph (Thermo Scientific, Waltham, MA, USA), using a standard mixture of fatty acid methyl esters (47885U) (Sigma, St. Louis, MO, USA) as the internal references.

#### 2.4.2. Vegetative Growth

Fungal radial growth was determined on CZA and SDAY plates. Conidial suspension (1 µL, 10^6^ conidia/mL) was inoculated on the plates. After 7 d of incubation at 25 °C, the colony diameter was examined. A feeding assay was conducted by adding oleic acid (0.3%) in medium. Oxidative stress was caused by including manedione (0.02 mM) into CZA plates. Conidial suspension was point inoculated on the plates and cultured at 25 °C.The colony diameter was determined at 7 d. Relative growth inhibition was calculated using the diameter on the CZA plate as a control.

#### 2.4.3. Conidial Production

An aliquot of conidial suspension (100 μL, 10^7^ conidia/mL) was uniformly smeared on SDAY plates and incubated at 25 °C for 9 d. From the sixth day after incubation, a mycelial disc (5 mm in diameter) was sampled, and conidia on the disc were washed into a 0.02% Tween-80 solution by vigorous vortexing. The conidial concentration of the suspension was quantified and used to calculate the conidial yield as the number of conidia per square centimeter. In the feeding assay, 0.3% oleic acid was added to the SDAY plates, and conidial production was also quantified as described above. Additionally, a transformant for the sub-cellular localization of BbSay1 was used to evaluate the effect of the over-expression of *BbSAY1* on conidiation.

#### 2.4.4. Conidia Germination

To simulate conidial germination under an oligotrophic condition, the conidial suspension was inoculated on a water agar plate (WA) (1.2% agarose). After an incubation of 24 h at 25 °C, the germination percentage was calculated. For each sample, at least 100 conidia were counted. A chemical feeding test was performed by adding oleic acid (0.3%) into the WA plates. To simulate conidial germination on the host cuticle, a conidial suspension (10^7^ cells/mL) was sprayed on the locust hindwings. After an incubation of 24 h at 25 °C, the conidial morphology was observed under a microscope.

#### 2.4.5. Insect Bioassay

To determine fungal virulence, the larvae of *Galleria mellonella* were used as the insect hosts. Last-instar larvae (~300 mg in weight) were used in this study [21]. In the trans-cuticular assay, larvae (30−35 individuals) were dipped in a conidial suspension (40 mL, 10^7^ conidia/mL) for 15 s. All infected insects were reared at 25 °C, and the mortality was recorded daily. The median lethal time (LT_50_) was calculated by a Probit analysis for three replicates of each bioassay. In the feeding test, oleic acid (0.3%) was added into the conidial suspension.

### 2.5. Lipidomic Analyses

The total lipid sample was prepared with the methods used in FA extraction as described previously [7]. Lipids in the sample were resolved and analyzed on UPLC-MS/MS (QE Plus™) (Thermo Scientific). A non-targeted lipidomics analysis platform combined with the Lipid Search™ software was applied for lipid identification and data processing. Data for the molecule intensity were plotted with the CV% method, which indicatedthe difference in the relative content in the total lipids between the WT and Δ*Bbsay1* mutant strains.

To view the variation of total lipids in mycelia, a conidial suspension was grown on the SDAY for 3 d at 25 °C. The resulting mycelia were stained with observed hyphae with the lipid-specific dye BODIPY493/503 (Thermo Fisher Scientific, Waltham, MA, USA).

### 2.6. Assay for Membrane Integrity

Nucleus staining with SYTOX Green was applied as previously described [7]. The conidia suspension was inoculated onto the SDAY plates and cultured for 12 h at 25 °C. The resultant germlings were collected and stained with SYTOX Green for 10 min away from light. The green fluorescence was detected under a laser scanning confocal microscope (LSM 710, Carl Zeiss Microscopy GmbH, Jena, Germany), and the percentage of stained cells (PSC) was calculated by counting at least 100 cells.

### 2.7. Data Analyses

All measurements from biochemical and phenotypic assays were subjected to a one-way ANOVA. The significant difference was determined by a Tukey’s honest significance test (Tukey’s-HSD).

## 3. Results

### 3.1. Bioinformatic Analysis and Molecular Manipulation of BbSay1

Based on the results of the BLAST search, the gene of BBA_02920 was significantly homologous to steryl acetyl hydrolase 1 in *S. cerevisiae* (GenBank No.: DAA08353) (E-value: 1 × 10^10^) and was designated as BbSay1. The open rearing frame of *BbSAY1* was 1453 bp in length with 3 introns, encoding a 386-amino acid protein. The domain analysis indicated that BbSay1 contained a domain of Say1_Mug180 (PF10340.9), and the domain annotation revealed that there were 10 Say1-domain-containing proteins in the *B. bassiana* genome. Their orthologs in other fungi were revealed via sequence alignment and domain annotation analyses (Table S3). Phylogenic analyses indicated that BbSay1 has a closer relationship to the orthologs from filamentous fungi than those of yeast species (Figure S1A). qRT-PCR analyses indicated that the Say1-domain protein genes displayed different expression patterns during growth in the nutrient medium. (Figure S1B). Under a fluorescent field, the green signals of the BbSay-Gfp protein distributed evenly in cytoplam (Figure S2). This indicated that BbSay1 dominated in the cytoplasm.

To uncover the physiological functions of BbSay1, its disruption mutant was constructed with a strategy of homologous replacement. To complement the gene loss, the entire ORF of *BbSAY1* and its promoter region was ectopically integrated in the genome of the Δ*Bbsay1* mutant strain. All candidate strains were screened, verified with PCR, and successfully confirmed by Southern blotting analyses (Figure S3).

### 3.2. Effects of Gene Loss on Fungal Phenotypes

**Figure 1:**
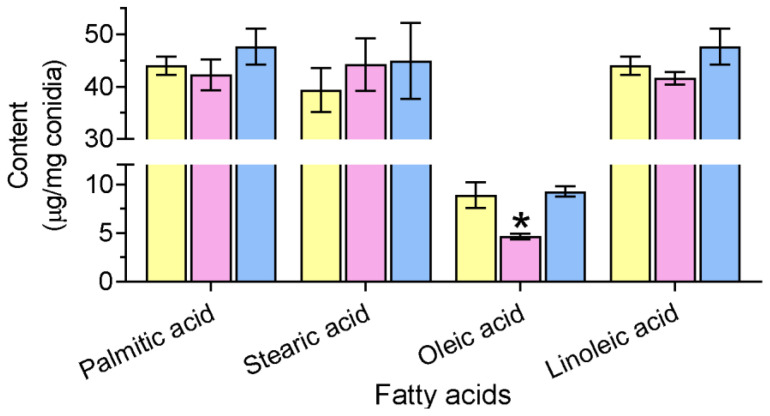
Fatty acid content in the *B. bassiana* conidia. The indicated strain was cultured on SDAY plates for 8 d, and the levels of fatty acids were determined in the conidia. The *B. bassiana* conidia accumulated four free fatty acids (stearic, palmitic, oleic, and linoleic acid) in the conidia. The gene disruption of *BbSAY1* led to a significant decrease in the level of oleic acid. The asterisk on the column indicates the significant difference in the fatty acid content between the Δ*Bbsay1* and the wild-type or complemented strains (Tukey’s HSD: *p* < 0.05). Error bars: standard deviation.

The *B. bassiana* conidia mainly accumulated four FAs, including stearic, palmitic, oleic, and linoleic acids. The ablation of *BbSAY1* resulted in a significant reduction in the content of oleic acid, and the reduction was 50.2%. However, the contents of the other three FAs were not significantly influenced (Figure 1).

**Figure 2:**
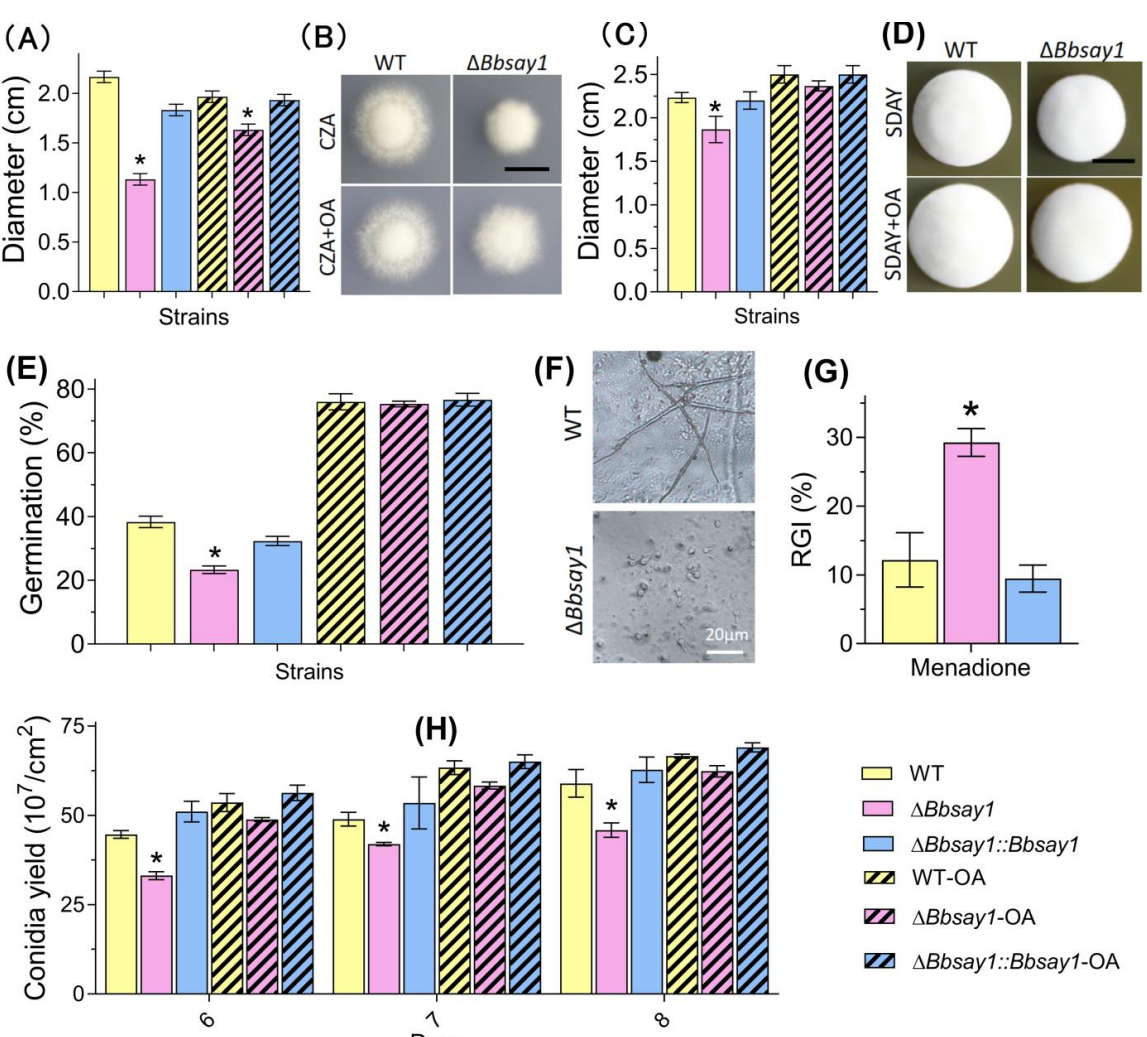
Fungal vegetative growth, germination, and conidiation. Fungal strains were inoculated on CZA (**A**) and SDAY (**C**) plates. After an incubation of 7 d at 25 °C, the colony diameter was examined. Photographs were taken for the colonies on the CZA (**B**) and SDAY (**D**) plates. Bars: 1 cm. (**E**) Conidial germination under an oligotrophic condition. The conidia were inoculated on a water agar plate (only with agarose) and cultured for 24 h at 25 °C, followed by determining the germination percentage. (**F**) Microscopic view of germlings on the hindwing of a locust. Bar: 20 μm. (**G**) Growth under oxidative stress. The conidial suspension was inoculated on a CZA plate plus menadione and cultured at 25 °C. Seven days later, the colony diameter was measured and used to calculate the relative growth inhibition (RGI). A CZA plate without manedione was used as a control. (**H**) Conidial production. Fugal strains were inoculated on SDAY plates and cultured at 25 °C. The conidial yield was examined at 6, 7, and 8 d post incubation. A chemical feeding assay was conducted by adding oleic acid (OA) to the indicated medium. The asterisks on the bars indicate the significant difference in the indicated phenotype between the Δ*Bbsay1* and the wild-type (WT) or complemented strains (Tukey’s HSD: *p* < 0.05). Error bars: standard deviation.

As shown in Figure 2A,C, the Δ*Bbsay1* mutant strain exhibit a significant decrease in colony size, with approximatelya 48.6% and 9.1% reduction on the CZA and SDAY plates, respectively, when compared with those of the wild-type (2.17 ± 0.05 cm (CZA) and 2.23 ± 0.05 cm (SDAY)) (mean ± standard deviation). Feeding oleic acid could increase the colony sizes on both the CZA and SDAY plates. This indicated that exogenous oleic acid could restore vegetative growth under an aerial condition. The colony morphologies on the CZA and SDAY plates areshown in Figure 2B and Figure 2D, respectively.

The conidial germination on WA plates was significantly decreased by gene disruption (Figure 2E). The germination percentage for the Δ*Bbsay1* mutant strain was 23.3 ± 2.1%; however, the wide-type and complementation strains displayed a germination percentage of approximately 35%. After adding oleic acid, there was no significant difference in the germination percentage among the wild-type, Δ*Bbsay1*, and complementation strains. On the hindwing of a locust, the conidia of the wild-type germinated well and developed into a germling; however, the Δ*Bbsay1* mutant displayed aretarded germination (F). Under oxidative stress by menadione, the gene disruption mutant displayed a significant defect in vegetative growth (G). The conidial yield was evaluated on SDAY plates (Figure 2H). Gene disruption caused a slight reduction in the conidial yield at three sampling time points. At 8 d post incubation, the conidial yield for the Δ*Bbsay1* mutant strain was 4.58 ± 0.35 × 10^8^ conidia/cm^2^, and that of the wild-type was 5.89 ± 0.67 × 10^8^ conidia/cm^2^. Exogenous oleic acid could reduce and eliminate this defect in the Δ*Bbsay1* mutant strain. A transformant with over-expression of *BbSAY1* generated 5.76 ± 4.48 × 10^8^ conidia/cm^2^, which did not significantly differ fromthe wild-type.

**Figure 3:**
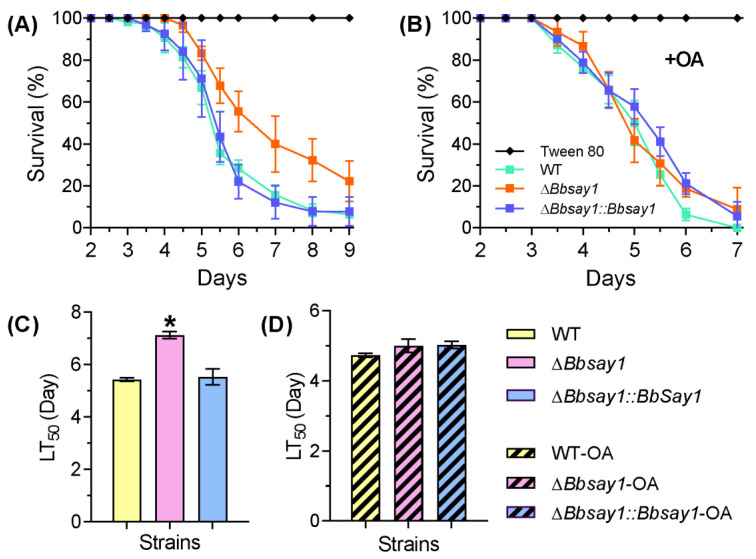
Fungal virulence. Conidial virulence was assayed for the wild-type (WT), Δ*Bbsay1,* and Δ*Bbsay1::Bbsay1* strains. The conidial suspension was flooded onto the host cuticle, and then, the inoculated insects were cultured at 25 °C. The survival trend was recorded daily (**A**), and the accumulative mortality was used to calculate the median lethal time (LT_50_) with a Probit analysis (**C**). In the feeding test, oleic acid (OA) was included in the conidial suspension, and accumulative mortality was used to calculate the survival trend (**B**) and LT_50_ (**D**). A Tween-80 solution was used as blank control. The asterisk on the column indicates the significant difference in the LT_50_ value between the Δ*Bbsay1* and the wild-type or complemented strains (Tukey’s HSD: *p* < 0.05). Error bars: standard deviation.

In a cuticle penetration bioassay, the survival trend for the Δ*Bbsay1* mutant showed a significant delay when compared to the wild-type (Figure 3A). The LT_50_ for Δ*Bbsay1* mutant was calculated as 7.13 ± 0.23 d (mean ± standard deviation), with a slight delay, when compared with that of the wild-type (5.43 ± 0.11 d) (Figure 3C). When adding oleic acid, there was no significant difference in the survival trend and LT_50_ between the wild-type and Δ*Bbsay1* mutant strains (Figure 3B,D).

### 3.3. Gene Loss Affects Cellular Lipidomics and Membrane Integrity

**Figure 4:**
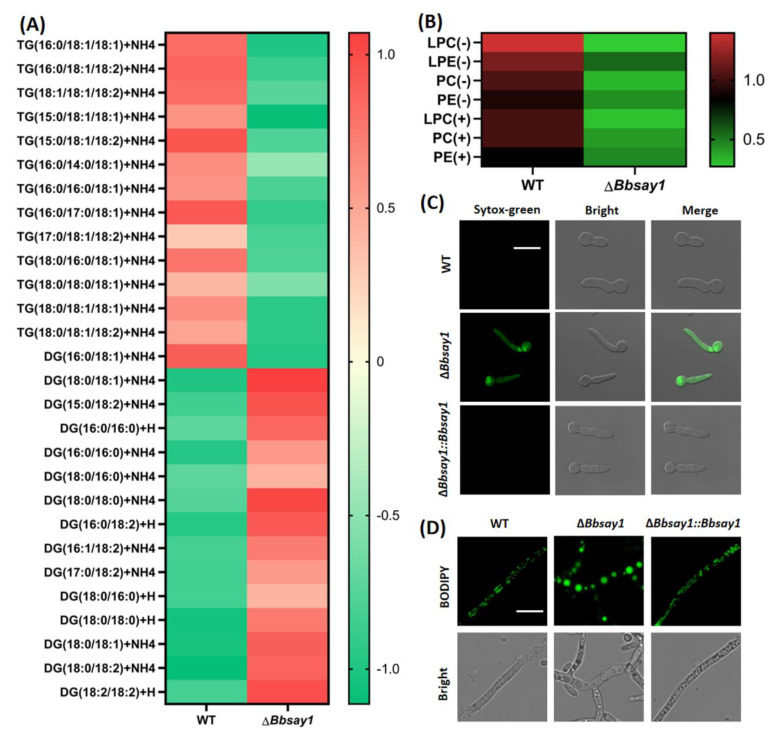
Assay for lipid homeostasis in *B. bassiana*. The lipidomic assay indicated that the ablation of *BbSAY1* resulted in the impaired homeostasis of a neutral lipid (**A**) and phospholipid (**B**). (**C**) Sytox-green nucleus staining. The Δ*Bbsay1* mutant strain displayed anincreased cytomembrane permeability, and its nuclei were easily stained by fluorescent dye. (**D**) Staining lipid bodies. The fluorescent dye BODIPY was used to stain the lipid bodies in fungal cells. The Δ*Bbsay1* mutant strain accumulated the enlarged lipid bodies, which indicatedthe unbalanced lipid metabolism after the ablating of *BbSAY1*.

As shown in Figure 4A, the ablation of *BbSAY1* resulted in an enhanced accumulation of diacylglycerols (DG) and a decrease in the content of triacylglycerols (TG). Four kinds of phospholipids were detected in the *B. bassiana* conidia, including lysophosphatidylcholine (LPC), lysophosphatidylethanolamine (LPE), phosphatidylcholine (PC), and phosphatidylethanolamine (PE), in their cationic or anionic forms. Gene disruption resulted in a significant decrease in the content of phospholipids (e.g., LPC) (Figure 4B). The loss of Bbsay1 led to poor membrane integrity in germlings. Without oleic acid, nearly all germlings were stained by SYTOX, while only 1% of the WT cells were stained (Figure 4C). Interestingly, exogenous oleic acids significantly reduced the percentage of the stained cells (PSC) in the Δ*Bbsay1* mutant strain. The microscopic view indicated that the size of lipid bodies was significantly increased when compared with that in the wild-type and complemented strains (Figure 4D).

## 4. Discussion

FA/lipid metabolism plays an important role in the interaction between entomopathogenic fungi and insect hosts [22]. This study has revealed that the ortholog of yeast steryl acetyl hydrolase 1 (Say1) contributes to lipid homeostasis, which is required for development and virulence in *B. bassiana*.

Lipid homeostasis is maintained by a series of biochemical reactions of esterification and transesterification, as well as the interaction among different catalytic pathways [9]. In *S. cerevisiae*, Say1 catalyzes the reaction of sterol deacetylation, which is required for sterol homeostasis [12,23]. In *B. bassiana*, BbSay1 contributes to the homeostasis of lipid metabolism. Its loss leads to a comprehensive fluctuation in the cellular lipidome, although more investigations are needed to reveal the direct effects of BbSay1 in *B. bassiana*. This finding suggests the conservative role of Say1 in lipid metabolism. It is well known that triacylglycerol is synthesized by the conjugation of acyl coenzyme A (acyl-CoA) to diacylglycerol, which is catalyzed by acyl-CoA:diacylglycerol acyltransferase [24]. After disrupting *BbSAY1*, *B. bassiana* accumulated a high level of diacylglycerol and a low level of triacylglycerol. Additionally, BbSay1 contributes to maintaining the morphologies of lipid bodies and the conidial reserve of oleic acid. Lipid droplets are organelles that store neutral lipids (i.e., triacylglycerols and steryl esters) and maintain the homeostasis of stored lipids and free fatty acids [25]. These results suggest that BbSay1 is further involved in lipid metabolism via modulating the biogenesis of lipid bodies. Considering the Say1 role in sterol homeostasis [23], our findings further suggest that there might exist an interaction between triacylglycerol and sterol homeostasis.

The conidial capacity to germinate and penetrate through the host cuticle is determinant for successful infection by a fungal entomopathogen [3]. Particularly, conidial germination on the nutrient-limited cuticle is critical for infection initiation [26]. Additionally, the cytomembrane functionality of germlings is critical for fungal virulence [7]. BbSay1 contributes to fungal virulence, which is due to its roles in conidial germination under the oligotrophic condition and the cytomembrane integrity. Several physiological processes have been linked to the cytomembrane integrity of the germling in *B. bassiana*, including the biosynthesis of unsaturated FA (e.g., BbOle1) [7]), lipid trafficking (e.g., BbScp2) [27], and thioesterification of FA (e.g., BbFaa1) [28]. In addition, a lectin-like protein functions at the interface between the cell membrane and wall and contributes to the cytomembrane functionality [29]. These findings reinforce that the homeostasis of lipid metabolism and the cell wall is essential for membrane integrity.

Conidia, generated through asexual development, are essential for entomopathogenic fungi to survive and disperse in the environment [14,30].BbSay1 contributes to conidiation in anoleic acid-dependent manner, and it maintains the morphology of lipid bodies during vegetative growth. In *A. nidulans*, two Δ9-stearic acid desaturases catalyze the biosynthesis of oleic acid, which is required for fungal development [31]. In filamentous fungi (e.g., *Blumeria* *graminis*), the oxidation of unsaturated fatty acids is involved in the conidiation process [32]. In *Magnaporthe* *oryzea*, the elevated accumulation of lipid bodies leads to severe conidiation defects [33]. Considering the roles of oleic acid in energy supply and the membrane integrity, our findings suggest that BbSay1 might affect multiple aspects in *B. bassiana* conidiation. In addition, BbSNF1, an AMPK protein kinase, mediates the amino-acid homeostasis during conidiation in *B. bassiana* [30]. These findings suggest that primary metabolism is essential for conidiation in filamentous fungi.

## 5. Conclusions

In conclusion, *B. bassiana* contains 10 Say1-domain-containing proteins. BbSay1, the ortholog of yeast Say1, maintains lipid homeostasis, which contributes to the cytomembrane integrity, development, and virulence in *B. bassiana*. In future, more investigations are needed to functionally identify other Say1 proteins, which should provide novel insights into the lipid metabolisms involved in the differentiation and virulence of entomopathogenic fungi.

## Data Availability

Not applicable.

## Supplementary Materials

The following supporting information can be downloaded at https://www.mdpi.com/article/10.3390/jof8030292/s1, Figure S1: Bioinformatic and transcriptional analyses. Figure S2: Sub-cellular localization of Say1 in *B. bassiana*. Figure S3: Molecular manipulation for gene function analysis of *B. bassiana*. Table S1: Primers for the qRT-PCR analyses. Table S2: Primers used for the gene disruption and complementation of *BbSAY1* in *B. bassiana*. Table S3: Homologs of *B. bassiana* Say1-domain-containing proteins in other fungal species.
