# Supplementary material for: SterylAcetyl Hydrolase 1 (BbSay1) Links Lipid Homeostasis to Conidiogenesis and Virulence in the Entomopathogenic Fungus Beauveria bassiana
Source: J Fungi (Basel). 2022 Mar 11;8(3):292. doi: 10.3390/jof8030292 (PMC8953178; doi:10.3390/jof8030292)

Figure S1 Bioinformatic and transcriptional analyses. (A) Phylogenetic relationship of BbSay1 with its homologs in yeasts and other filamentous fungi. Relationships among proteins were depicted from Neighbor joining analysis and the bootstrap values > 50% from 1000 replicates are shown as numbers at each supported branch. (B) Expression profiles for the Say1-domain protein genes in *B. bassiana*. The wild-type strain was cultured on SDAY plate and sampled at 2, 3, 4, and 5 d. The relative expression levels were normalized to that at 2 d and shown in a hotmap.

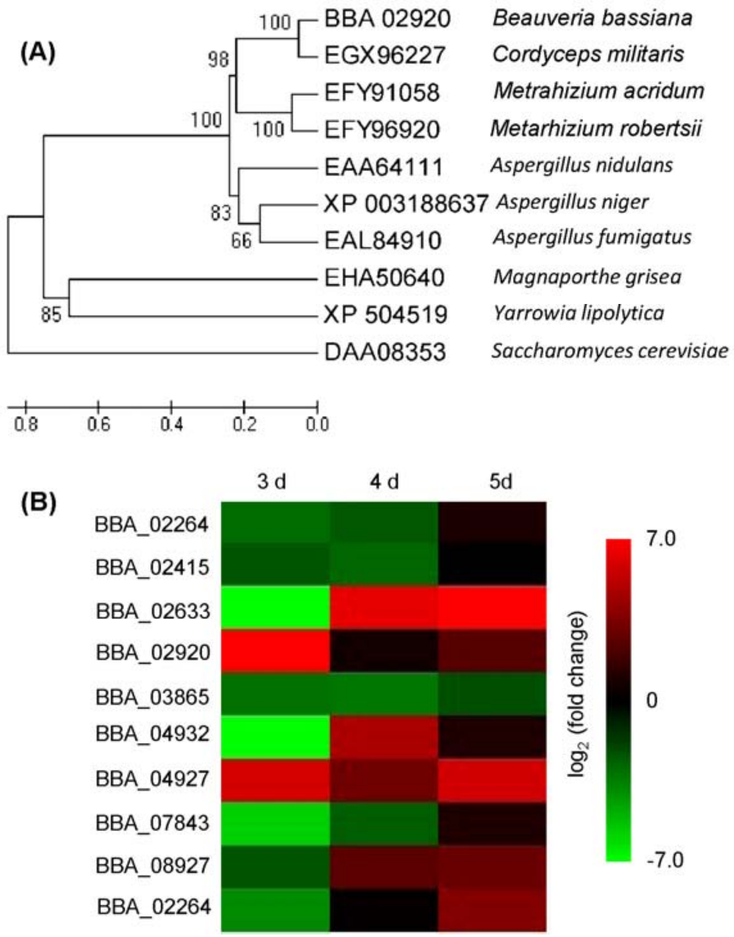

Supplement: Supplementary file 1 [file jof-08-00292-s001.zip › Figure S1.pdf]
