# Supplementary material for: SterylAcetyl Hydrolase 1 (BbSay1) Links Lipid Homeostasis to Conidiogenesis and Virulence in the Entomopathogenic Fungus Beauveria bassiana
Source: J Fungi (Basel). 2022 Mar 11;8(3):292. doi: 10.3390/jof8030292 (PMC8953178; doi:10.3390/jof8030292)

Figure S2 Sub-cellular localization of Say1 in *B. bassiana*. *BbSAY1* was fused to green fluorescent protein gene (*GFP*) and transformed into the wild-type strain. The resultant strain was cultured on SDAY plate and the fluorescent signals in mycelia were observed under a laser scanning confocal microscope. Bar: 5 μm.

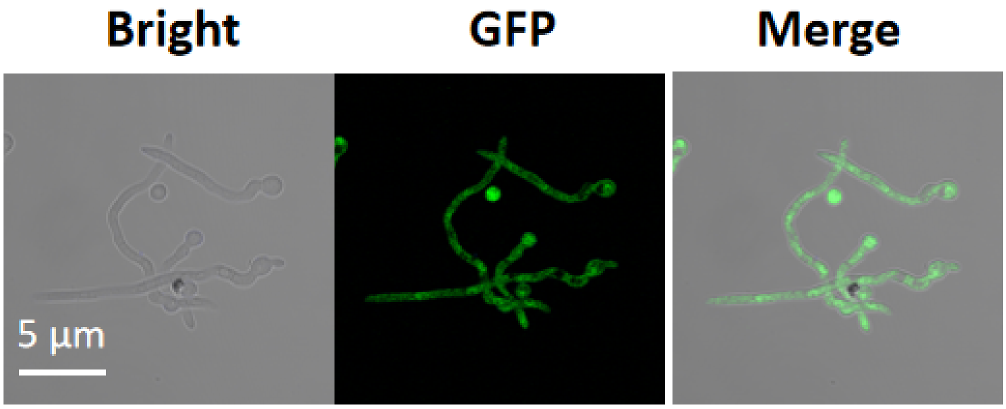

Supplement: Supplementary file 1 [file jof-08-00292-s001.zip › Figure S2.pdf]
