# Supplementary material for: SterylAcetyl Hydrolase 1 (BbSay1) Links Lipid Homeostasis to Conidiogenesis and Virulence in the Entomopathogenic Fungus Beauveria bassiana
Source: J Fungi (Basel). 2022 Mar 11;8(3):292. doi: 10.3390/jof8030292 (PMC8953178; doi:10.3390/jof8030292)

Figure S3 Molecular manipulation for gene function analysis of *B. bassiana*. (A) Schematic diagram of gene disruption in fungal genome using homologous recombination strategy. Candidate transformants were screened by PCR (B) and further confirmed by Southern blot analysis (C). In Southern blotting, the enzyme pair *Sac*I/*Nco*I was used to digest genomic DNA. The numbers near the images in (B) and (C) indicate the molecular sizes of DNA fragments. Lane 1: wild type; lane 2: Δ*Bbsay1*; lane 3: Δ*Bbsay1::Bbsay1*; lane M: molecular marker.

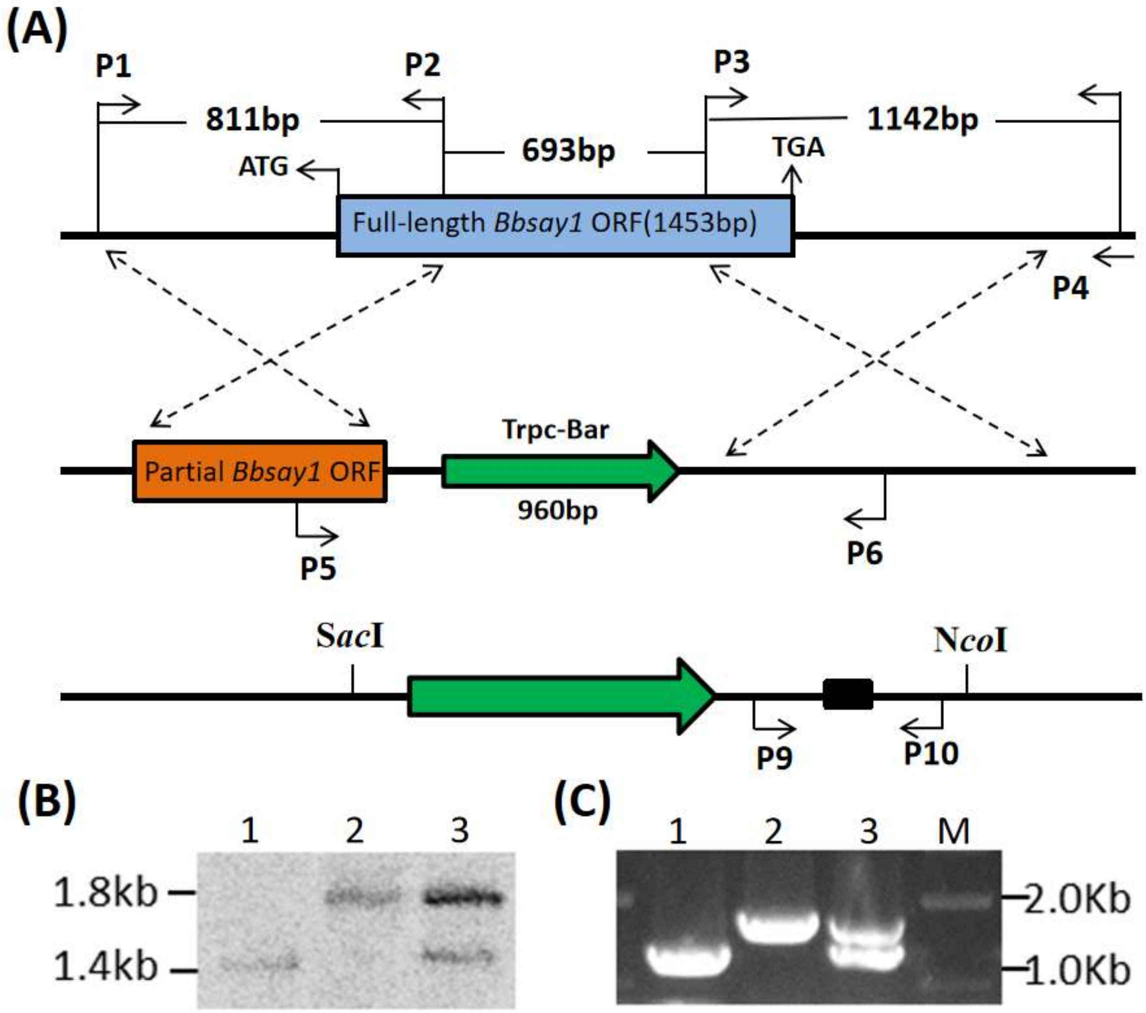

Supplement: Supplementary file 1 [file jof-08-00292-s001.zip › Figure S3.pdf]
