# Supplementary material for: SterylAcetyl Hydrolase 1 (BbSay1) Links Lipid Homeostasis to Conidiogenesis and Virulence in the Entomopathogenic Fungus Beauveria bassiana
Source: J Fungi (Basel). 2022 Mar 11;8(3):292. doi: 10.3390/jof8030292 (PMC8953178; doi:10.3390/jof8030292)
Supplement: Supplementary file 1 [file jof-08-00292-s001.zip › Table S1.pdf]

Table S1 Primers for qRT-PCR analyses.

Primer name	Sequence (5′–3′) [a]	Gene for qPCR
P1F	GTGTCTTTGGGGTAGAGGGC	BBA_04392
P1R	CCTCCGTAATACCTGACCGC	
P2F	GGTTGCCGCCATTATTCGTC	BBA_03865
P2R	CGCATATTTCTGCGGGTTGG	
P3F	TAAAGGCCGGTCATCCCAAC	BBA_02920
P3R	TGCTCCGTCTCTGTGTTGTC	
P4F	TTTGGCGTCCTCACCTACAC	BBA_02633
P4R	TGGAAGCGAACCCGCTTATT	
P5F	GTTTCTTGCCGCGTGACATT	BBA_02415
P5R	TCGTTGTTGCCAAAGATGCG	
P6F	CCCGAGAAAACAGAGGGCAT	BBA_02264
P6R	AAAACTGCAGCGAAACGACC	
P7F	TATCGACGCTTCGTCTCTGC	BBA_07843
P7R	GAAGCAGCTCCCCTCCAAAT	
P8F	CAAACGCAACCTCCAAAGGG	BBA_09015
P8R	TCTTGGATGAGGCTCTCGGA	
P9F	TGGATCTCATCCCAGGAGCA	BBA_08937
P9R	CAACGTGCGCTTTGTTGGTA	
P10F	CCCACTATGGCGTGTATCCC	BBA_04927
P10R	CAGGTCGAGCTGTCCAACAT	
18Sf	TGGTTTCTAGGACCGCCGTAA	Reference
18Sr	CCTTGGCAAATGCTTTCGC	
