# Supplementary material for: SterylAcetyl Hydrolase 1 (BbSay1) Links Lipid Homeostasis to Conidiogenesis and Virulence in the Entomopathogenic Fungus Beauveria bassiana
Source: J Fungi (Basel). 2022 Mar 11;8(3):292. doi: 10.3390/jof8030292 (PMC8953178; doi:10.3390/jof8030292)
Supplement: Supplementary file 1 [file jof-08-00292-s001.zip › Table S2.pdf]

**Table S2 Primers used for gene disruption and complementation of *BbSAY1* in *B. bassiana***

| Primer name | Sequence (5′–3′) [a] | Purpose of use |
|---|---|---|
| PL1 | TCAAACCCGGGGATATCATGGCAACCTCAACCCGAA | Amplifying *BbSAY1* |
| PL2 | CTTGCTCACCATGTTAACTTTCTGCAATTCAATCAG | |
| $P_1$ | TGGGCCCGGCGCGCCGAATTCCAGGATGCTGTGCCAGATAG | Obtaining upstream flanking sequence |
| $P_2$ | TGGCTGCAGGTCGACGGATCCCTATTTACGAACGCCTCCTG | |
| $P_3$ | GACCCATGGCTCGAGTCTAGACACGGAGCCCAAGGACAT | Obtaining downstream flanking sequence |
| $P_4$ | GGTGGTGGTGGCTAGCGTTAACCGATCTTCCGCTCCGACT | |
| $P_5$ | TCCGACTGTTGATTGTGCTG | Confirming the candidate transformants |
| $P_6$ | CATTCTTGTTGCCCACCC | |
| $P_7$ | ATCCGTCGACCTGCAGCCAAGCTTCGCTCCTCGAGATTCTGG | Amplifying *Lip1* for complementation |
| $P_8$ | ACACTAGAAGATCTGACTAGTGTGCTTGCCATCATCAATAAAC | |
| $P_9$ | GGGTGGGCAACAAGAATG | Probe preparing in Southern blot |
| $P_{10}$ | GAGTCGCCGCCGAG | |
