# Supplementary material for: SterylAcetyl Hydrolase 1 (BbSay1) Links Lipid Homeostasis to Conidiogenesis and Virulence in the Entomopathogenic Fungus Beauveria bassiana
Source: J Fungi (Basel). 2022 Mar 11;8(3):292. doi: 10.3390/jof8030292 (PMC8953178; doi:10.3390/jof8030292)
Supplement: Supplementary file 1 [file jof-08-00292-s001.zip › Table S3.pdf]

Table S3 Homologs of *B. bassiana* Say1-domain-containing proteins in other fungal species.

BBA[*,#]	AND[†]	ANI	AFU	CAL	SCE	YLI	CMI	MAC	MRO	MGR
BBA_02264	EAA63899	XP_003188637	EAL91622	XP_711433	NA	XP_504519	EGX89271	EFY91356	EFZ00850	EHA50963
BBA_02415	EAA65442	XP_001400753	EAL84910	XP_711433	NA	NA	EGX96126	EFY93198	EFZ01185	EHA50640
BBA_02633	EAA61114	XP_001401604	EAL88803	XP_721919	NA	NA	EGX90144	EFY86198	NA	EHA48364
BBA_02920	EAA64111<br>42.5%[¶]	XP_003188637<br>44.3%	EAL84910<br>45.5%	NA	DAA08353<br>14.9%	XP_504519<br>13.8%	EGX96227<br>78.4%	EFY91058<br>54.1%	EFY96920<br>54.9%	EHA50640<br>19.3%
BBA_03865	NA	XP_001394739	EAL88803	XP_721834	NA	XP_504071	EGX90887	NA	EFZ02604	EHA48364
BBA_04392	EAA65442	XP_001395501	EAL84910	XP_710482	NA	NA	EGX90339	EFY86198	KHO11046	EHA50640
BBA_04927	NA	NA	NA	XP_721919	NA	NA	NA	NA	NA	NA
BBA_07843	EAA65442	XP_001392749	EAL87524	XP_721919	NA	NA	EGX96227	EFY86198	EFZ01185	EHA50640
BBA_08937	EAA64111	XP_003188637	EAL84910	NA	NA	NA	EGX96227	EFY91058	EFZ03052	EHA50640
BBA_09015	EAA65442	XP_003188637	EAL87524	NA	NA	NA	EGX96227	EFY86198	KHO11046	EHA50640

*: abbreviations for fungal species

BBA: *Beauveria bassiana*; AND: *Aspergillus nidulans*; ANI: *A. niger*; AFU: *A. fumigatus*; YLI: *Yarrowia lipolytica*; SCE: *Saccharomyces cerevisiae*; CAL: *Candida albicans*; MGR: *Magnaporthe grisea*; CMI: *Cordyceps militaris*; MRO: *Metarhizium robertsii*; MAC: *M. acridum*.

#:*B. bassiana* Say1-domain-containing proteins are indicated with their numbers of locus tag.

†: The homologs in other fungi are indicated with their accession numbers in GenBank.

¶: The amino acid identity (%) between BbSay1 and its orthologs in other fungi.

NA: No homolog of *B. bassiana* is present in the indicated fungal species.
